# Nanoplatform‐Enabled Genetic Interventions for Central Nervous System Disorders: Advances in Delivery Strategies and Therapeutic Potential

**DOI:** 10.1002/ggn2.202500010

**Published:** 2025-06-24

**Authors:** Fuming Liang, Shizhen Cui, Jing Yang, Zhaohui He, Ling Zhu

**Affiliations:** ^1^ CAS Key Laboratory of Standardization and Measurement for Nanotechnology National Center for Nanoscience and Technology Beijing 100190 P. R. China; ^2^ Department of Neurosurgery The First Affiliated Hospital of Chongqing Medical University 1 Friendship Road Chongqing 400016 P. R. China; ^3^ Chengdu Medical College Chengdu 610500 P. R. China; ^4^ Institute of Blood Transfusion Chinese Academy of Medical Sciences Peking Union Medical College Chengdu 610052 P.R. China; ^5^ University of Chinese Academy of Sciences Beijing 100049 P. R. China

**Keywords:** blood brain barrier, central nervous system diseases, genetic therapy, nanocarrier, targeted delivery

## Abstract

Central nervous system (CNS) disorders are driven by complex genetic and epigenetic factors. While gene‐based interventions (siRNA, mRNA, CRISPR systems, etc.) hold transformative potential, their clinical application is severely constrained by inefficient delivery, especially across the blood‐brain barrier. Nanocarriers have emerged as transformative platforms that overcome these challenges by enabling efficient BBB penetration while ensuring precise biodistribution control and enhanced therapeutic payload protection. This review explores recent advances in nanoplatform‐enabled genetic intervention that overcome the delivery challenges through innovative engineering approaches. We discuss the genetic and epigenetic mechanisms underlying major CNS pathologies, the current limitations of free nucleic acid therapeutics, the development of advanced nanoplatforms that achieve blood‐brain barrier penetration and targeted delivery. We further also evaluate therapeutic prospects across disease models while addressing translational challenges in stability, targeting specificity, and manufacturing scalability. By integrating fundamental research with preclinical applications, this review provides both a theoretical framework and practical roadmap for developing next‐generation nanotherapeutics for CNS genetic medicine.

## Graphical abstract

This review provides a detailed description of gene mutations and epigenetic mechanisms in  central nervous system (CNS) disease, strategies for gene therapy, challenges for CNS gene therapy and gene‐drug nanocarriers. This review also summarizes the applications of nanogenetic therapy platforms in CNS diseases (brain tumors, neurodegenerative diseases, ischemic stroke, and traumatic brain injury).

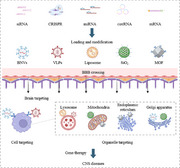

## 1 Introduction

Brain diseases include brain tumors, neurodegenerative diseases, ischemic stroke, and traumatic brain injury. The occurrence, development, and prognosis of these CNS diseases are closely related to genetic mutations and epigenetic regulations.^[^ ^1^ ^]^ Mutations in nuclear and mitochondrial genes mainly include point mutations, insertion or deletion mutations, small segmental duplications, large segmental mutations, and dynamic mutations.^[^ ^2^ ^]^ These mutations lead to the creation of pathological new genes or the inactivation of physiological genes that induce central nervous system (CNS) disease. For example, mutations in the isocitrate dehydrogenase 1 (IDH1) gene lead to intracellular accumulation of 2‐hydroxyglutarate (2‐HG) and induce glioblastoma (GBM).^[^ ^3^ ^]^ In addition to genetic mutations, abnormal epigenetic regulation is also an important factor in CNS disease development. Epigenetic regulation is the chemical modification of genes or gene products, including methylation of DNA or RNA and regulatory effects of non‐coding RNAs (miRNAs, lncRNAs, circRNAs).^[^ ^4^ ^]^ For example, high expression of methylated reader YTHDF2 recognizes METTL3‐mediated m6A modification, which activates the NF‐κB signaling pathway and promotes the malignant progression of GBM.^[^ ^5^ ^]^ Currently, in response to mutations and abnormal epigenetic regulation, researchers have designed a range of gene drugs to perform interventions. For example, siRNAs,^[^ ^6^ ^]^ miRNAs,^[^ ^7^ ^]^ mRNAs,^[^ ^8^ ^]^ circRNAs,^[^ ^9^ ^]^ and CRISPR gene editors.^[^ ^10^ ^]^

However, gene therapy for CNS currently faces two major difficulties. First, gene drugs (e.g., siRNA, miRNAs, mRNA, circRNA, CRISPR, etc.) are easily cleared in the circulation and difficult to reach the CNS.^[^ ^11^ ^]^ Second, the blood‐brain barrier (BBB) is a natural protective barrier for the CNS and hinders the accumulation of gene drugs in the CNS.^[^ ^12^ ^]^ These two major difficulties severely limit the efficacy of gene drugs for CNS disease. Therefore, finding new gene‐drug carriers is important to improve the efficiency of gene therapy for CNS diseases. Nanomaterials are a class of multifunctional, small‐sized, and drug‐loading carriers that can load gene drugs through electrostatic adsorption, electroporation, and chemical bonding.^[^ ^13^ ^]^ Currently, the main delivery nanomaterials commonly used for gene drugs include biomimetic nanovesicles (BNVs), virus‐like particles (VLPs), protein nano drug delivery platforms, liposomes, polymeric nanocarriers and some inorganic nanomaterials. These nanomaterials are structurally stable, not susceptible to clearance by blood mononuclear macrophages, and can be surface‐modified to promote the crossing of the BBB. For example, researchers synthesized REXO‐C/ANP/S by loading siSNCA into small extracellular vesicles of rabies virus glycoprotein (RVG)‐modified macrophages.^[^ ^14^ ^]^ RVG and macrophage vesicles achieve BBB crossing and gene‐drug escape from blood clearance, respectively, thus enhancing therapeutic efficacy in neurodegenerative diseases. Therefore, nanomaterials are ideal carriers for gene drugs and enable the aggregation of gene drugs at the CNS site to improve their therapeutic efficiency.

**Figure 1:**
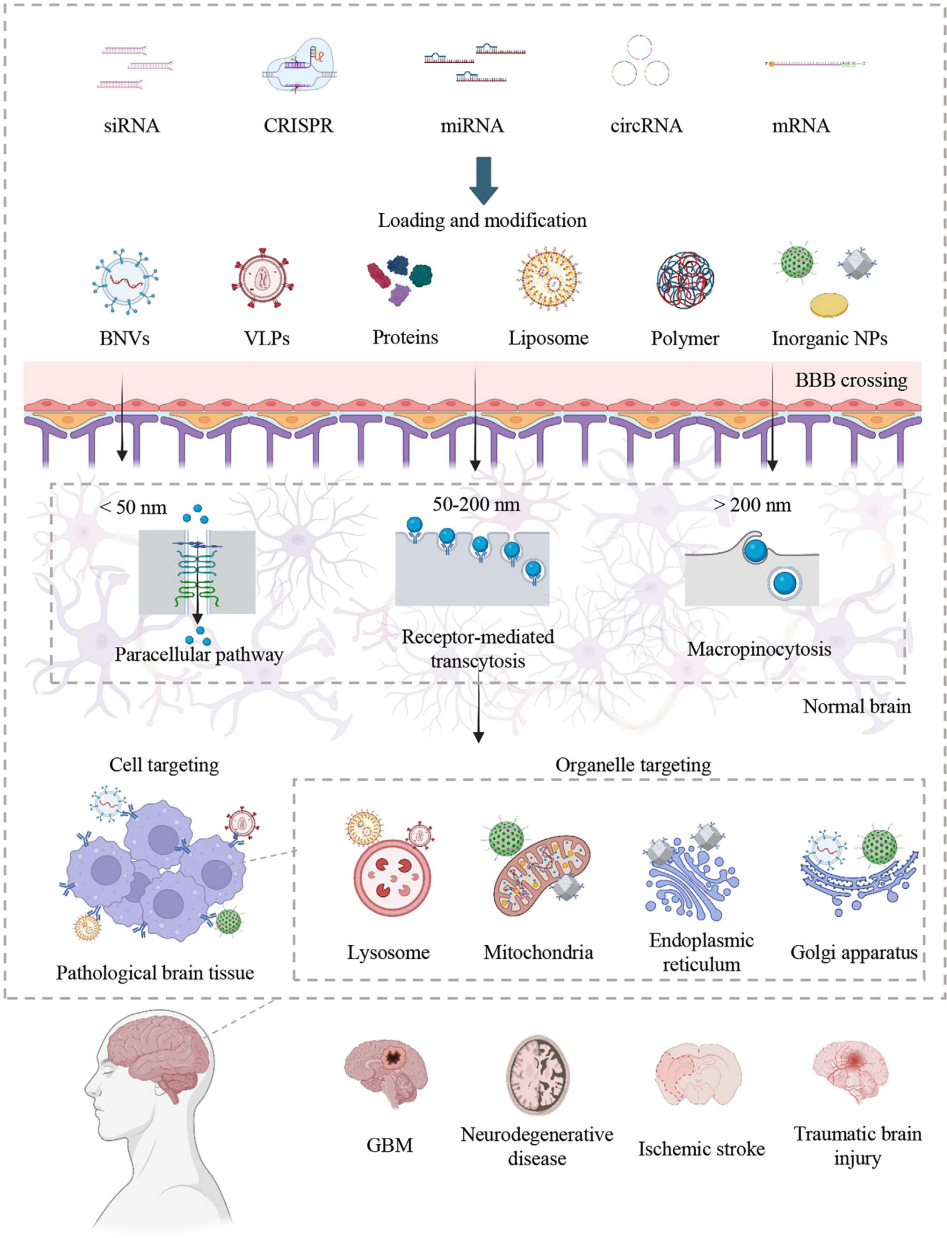
Nanoplatforms for CNS‐targeted genetic therapy. Nanoplatforms such as bioinspired nanovesicles (BNVs), virus‐like particles (VLPs), protein‐based carriers, liposomes, polymeric nanoparticles (NPs), and inorganic NPs are utilized for targeted delivery of genetic therapeutics to the central nervous system (CNS). These platforms efficiently transport siRNA, CRISPR‐Cas systems, miRNAs, circRNAs, and mRNAs across the blood‐brain barrier (BBB) via multiple mechanisms, such as paracellular transport, receptor‐mediated transcytosis, and micropinocytosis. They also exhibit remarkable capabilities in lesion‐specific targeting, selective cell population engagement, and even subcellular organelle localization. Such targeted delivery systems hold significant therapeutic potential for a range of CNS disorders, including glioblastoma (GBM), neurodegenerative diseases (e.g., Alzheimer's and Parkinson's), ischemic stroke, and traumatic brain injury (TBI).

This review systematically discusses the role of genetic mutations and epigenetic dysregulation in CNS pathologies, while evaluating contemporary gene therapy strategies and their prospects and limitations in CNS disorder treatment. We highlight cutting‐edge advances in nanoplatform‐engineered carriers designed for BBB penetration and cell‐specific targeting, which address critical challenges in nucleic acid delivery. We further summarize the applications of these nanotechnologies across diverse CNS disorders—including brain tumors, neurodegenerative diseases, ischemic stroke, and traumatic brain injury (**Figure** **1**). By unifying fundamental principles with translational considerations, we provide a conceptual framework and actionable strategies for advancing next‐generation genetic nanomedicines for CNS disorders.

## 2 Genetic Markers for CNS Disorders

Genetic markers of CNS diseases mainly include a range of pathological genetic material produced by nuclear mutations, mitochondrial mutations, and altered epigenetic modifications.^[^ ^15^ ^]^ These substances promote the development of GBM and neurodegenerative diseases and negatively affect the prognosis of ischemic stroke and traumatic brain injury.

### 2.1 Nuclear Gene Mutation

Mutations in nuclear genes mainly include point mutations, insertion or deletion mutations, small segmental duplications, large segmental mutations, and dynamic mutations. These mutations lead to the alterations of gene function or the loss of existing genes, which can induce a variety of CNS diseases. For example, mutations in the IDH1 gene of GBM lead to the accumulation of 2‐HG in vivo. 2‐HG is a metabolite that affects the methylation patterns of DNA and histones, thereby disrupting the epigenetic state of cells.^[^ ^16^ ^]^ In addition to brain tumors, genetic mutations can lead to misfolding and abnormal accumulation of pathological proteins, which can lead to neurodegenerative diseases. For example, mutations in the SNCA gene cause the core hydrophobic region of α‐Synuclein to form a β‐sheet structure, which promotes fibrosis and accumulation and ultimately leads to the development of PD.^[^ ^14^ ^]^

### 2.2 Mitochondrial Gene Mutation

Mitochondrial DNA (mtDNA) is primarily responsible for encoding certain proteins in the mitochondria that are involved in the cell's oxidative phosphorylation process, a key step in the cell's energy production. Mutations in mtDNA are also involved in the CNS disease process. For example, PITRM1 acts predominantly as a mitochondrial matrix enzyme in cells and is responsible for the degradation of short unstructured peptides as well as various forms of amyloid β‐peptide. Mutations in the PITRM1 gene cause excessive deposition of amyloid and promote AD pathology.^[^ ^17^ ^]^

### 2.3 Epigenetics

Epigenetic changes do not involve alterations in the DNA sequence, but rather chemical modifications to regulate gene activity. Epigenetics mainly includes DNA or RNA methylation and non‐coding RNA regulation (miRNAs, lncRNAs, circRNAs).

DNA or RNA methylation is the addition of methyl groups to specific positions in the molecule. This process is regulated by multiple molecules and catalyzed by enzymes. For example, m6A methylation is a chemical modification found on RNA molecules, which is the process of adding a methyl group (‐CH3) to the adenine N6 position in the RNA molecule. The m6A methylation modification is a reversible process jointly regulated by methylase (METTL3), demethylase (ALKBH5), and methylated reading protein (YTHDF1, YTHDF2, CENPA).^[^ ^18^ ^]^ Here, we focus on the role of different methylated reading proteins. YTHDF1 promoted translation of m6A‐modified mRNAs and is overexpressed in cancers like gastric cancer. Targeting YTHDF1 via siRNA delivered by engineered sEV has shown efficacy in suppressing tumor growth by disrupting Wnt/β‐catenin signaling and enhancing immune responses (e.g., IFN‐γ signaling and MHC‐I presentation).^[^ ^19^ ^]^ Abnormal expression of these enzymes that regulate m6A methylation will promote CNS diseases. In GBM, high expression of methylated reader YTHDF2 recognizes METTL3‐mediated m6A modification, which activates the NF‐κB signaling pathway to promote the malignant progression of GBM.^[^ ^5^ ^]^ In addition, recent studies reveal that CENPA, an m6A reader localized at centromeres, stabilizes m6A‐modified cenRNAs to ensure centromere integrity in cancer cells. Disrupting CENPA‐cenRNA interactions induces chromosomal instability, impairing tumor proliferation and offering a potential strategy for cancer therapy.^[^ ^20^ ^]^

Non‐coding RNA regulation is mainly mediated by miRNAs, lncRNAs, and circRNAs. miRNAs mediate post‐transcriptional gene silencing by binding to the 3′ untranslated or open reading frame regions of target mRNAs, thereby regulating gene expression. LncRNAs can act as sponges for miRNAs and regulate gene expression by pairing with miRNA complementary sites and reducing the targeting of miRNAs to mRNAs. circRNAs can bind to miRNAs and affect miRNA activity, thereby regulating the expression of target mRNAs. Changes in the expression of these non‐coding RNAs induce the pathology of CNS disease. For example, miRNA‐26 and miRNA‐21 mediated GBM angiogenesis; miRNA‐10 mediated immune escape of GBM cells; circ‐MMP1 mediated tumor invasion and metastasis; and lnc‐CCAT2 mediated neuroinflammation.^[^ ^21^ ^]^ Therefore, targeting non‐coding RNAs is also an important way for the treatment of CNS diseases.

The interplay between epigenetic regulation and drug delivery has emerged as a pivotal area in advancing precision medicine, particularly in oncology. Epigenetic modifications, including DNA or RNA methylation and non‐coding RNA‐mediated regulation, not only drive tumorigenesis and drug resistance but also offer novel targets for therapeutic intervention.^[^ ^18^ ^]^ Recent advancements in drug delivery systems have enabled precise targeting of epigenetic modulators, overcoming limitations such as off‐target effects and systemic toxicity. For example, the integration of epigenetic drugs with immune checkpoint inhibitors has shown promise. Low‐dose DNA methyltransferase inhibitors (e.g., decitabine) enhance chemosensitivity in bladder cancer by targeting cancer stem cells, while HDAC inhibitors upregulate MHC‐I expression, improving T‐cell recognition.^[^ ^22^ ^]^ Such strategies highlight the dual role of epigenetic regulators in both reprogramming tumor cells and amplifying immune responses.

## 3 Strategies for CNS Genetic Therapy

The main strategy of CNS genetic therapy is to deliver gene drugs to the lesion site for overexpression, knockdown, or editing of target genes. The common CNS gene therapy drugs mainly include siRNAs, CRISPR gene editing systems, miRNAs, circRNAs, and mRNAs. Here we will focus on the working mechanism of each gene‐drug and its application in CNS diseases (**Table** **1**).

**Table 1:** The representative applications, Challenges and limitations of CNS gene therapy strategies.

Strategies	Mechanisms	Target genes	CNS diseases	Challenges and limitations	References
siRNA	Silencing of mRNA	RBBP4	GBM	Stability and nuclease degradation; endosomal escape; off‐target effects	[24]
CRISPR	Knockout of the target gene, base editing, or degradation of the RNA	MDK	GBM	Large payload size; immunogenicity; off‐target editing; nuclear delivery	[26]
miRNA	Degradation of the mRNAs	miR‐9, miR‐21, miR‐215, and miR‐221	GBM	Dual roles and context dependency; delivery precision; stability and dosage	[27]
circRNA	Binding miRNA	SCMH1	Ischemic stroke	Production complexity; immune activation; functional persistence	[29]
mRNA	Direct expression of target proteins	PTEN	GBM	Innate immune activation; cold chain dependency; transient expression	[31]

### 3.1 siRNA

Small interfering RNA (siRNA)‐mediated gene therapy represents a groundbreaking approach in the field of molecular medicine, offering a highly specific and potent means of silencing disease‐causing genes. siRNAs are short, double‐stranded RNA molecules, typically 20–25 nucleotides in length, that guide the RNA‐induced silencing complex (RISC) to complementary mRNA targets, resulting in their degradation and subsequent inhibition of protein synthesis.^[^ ^23^ ^]^ This mechanism, known as RNA interference (RNAi), has been harnessed for therapeutic purposes to target a wide range of genetic disorders, viral infections, and cancers. In CNS diseases, silencing of target genes by siRNA is widely used and highly effective. For example, In a study, temozolomide, siRBBP4, and the brain‐targeting peptide Ang self‐assembled to synthesize APS@(siRNA&TMZ). APS@(siRNA&TMZ) crossed the BBB under the effect of Ang peptide and downregulated MGMT expression after silencing the RBBP4 gene. This effect inhibited temozolomide‐associated DNA damage repair, thereby increasing the sensitivity of GBM cells to temozolomide and providing a synergistic therapeutic effect.^[^ ^24^ ^]^

### 3.2 CRISPR System

The CRISPR gene editing system uses site‐specific Cas nuclease to introduce DNA or RNA breaks in the presence of guide RNA, and then repairs the breaks by the cell's own non‐homologous end joining (NHEJ) or homologous recombination repair (HDR), ultimately leading to the knockout of the target gene, base editing, or degradation of the RNA.^[^ ^25^ ^]^ Depending on the composition of the CRISPR gene editing system, different biological functions are performed, for example, cas9 is mainly used to target DNA while cas13 is mainly used to target RNA. CRISPR gene editing system holds broad promise for use in CNS disease. For example, researchers designed a reactive oxygen species (ROS)‐responsive lipid nanoparticle loaded with a CRISPR cas9 system for editing the MDK gene in GBM cells. This gene editing system unlocks the lock that restricts drug release in response to a ROS environment, thereby allowing lipid nanoparticles to release the CRISPR cas9 system into GBM cells via membrane fusion. In the presence of MDK guide RNA, CRISPR cas9 accurately recognized the MDK gene and knocked it out, resulting in significant inhibition of GBM growth.^[^ ^26^ ^]^

### 3.3 miRNA

MicroRNAs (miRNAs) are small, non‐coding RNA molecules, typically 20–24 nucleotides in length, that play a critical role in post‐transcriptional regulation of gene expression. The mechanisms in which miRNAs play a regulatory role mainly involve translational repression and mRNA degradation. miRNAs can bind to the 3′UTR of mRNAs, resulting in the recognition and degradation of the mRNAs by nuclease enzymes in the cell, thus reducing the translation of target genes. In CNS disease, higher transcriptional activity of some genes induces the pathological process of the diseases, so inhibiting the translational expression of target genes by delivering miRNAs is promising to make progress in the treatment of CNS disease. For example, investigators constructed a BV2 cell membrane‐encapsulated miRNAs nanosponge that adsorbed miRNAs in GBM cells, thereby inhibiting malignant growth and invasion of GBM mediated by miR‐9, miR‐21, miR‐215, and miR‐221.^[^ ^27^ ^]^

### 3.4 circRNA

Circular RNA (circRNA) is an endogenous RNA with a covalently closed loop structure. circRNA has higher stability and longer half‐life compared to linear RNA because it is less susceptible to degradation by nucleic acid exonucleases.^[^ ^28^ ^]^ CircRNAs are rich in miRNA binding sites, which can adsorb miRNAs in cells, thereby relieving the inhibitory effect of miRNAs on target genes. In CNS disease, cirRNA‐mediated gene therapy promotes repair of brain damage and clearance of neuroinflammation. Researchers synthesized circSCMH1‐EV‐RVG nanomaterials by loading CircSCMH1 in 293T cell‐derived sEVs and modifying the brain‐targeting peptide RVG. circSCMH1‐EV‐RVG targets ischemic stroke sites in the presence of RVG, and the released circSCMH1 promotes neuronal axonal regeneration, reduces infiltration of inflammatory cells in the brain, and transforms microglia into an anti‐inflammatory phenotype.^[^ ^29^ ^]^

### 3.5 mRNA

mRNA‐mediated gene therapy is an emerging therapeutic approach that utilizes messenger RNA (mRNA) as a carrier to deliver genetic information encoding specific proteins into the cell so that the target proteins can be expressed within the cell. The advantage of this therapy is that mRNA does not need to enter the nucleus and can express proteins directly in the cytoplasm for rapidly dividing or non‐dividing cells.^[^ ^30^ ^]^ In CNS diseases, the growth of GBM is caused by the inactivation of oncogenes, so prompting the expression of oncogenes in tumor cells by delivering their mRNAs will give hope for the treatment of GBM. For example, researchers synthesized ABNPs@mRNA nanomaterials by loading the mRNA of the oncogene PTEN into erythrocyte membranes and modifying apolipoprotein E (ApoE) on the surface. ApoE on ABNPs@mRNA binds to low‐density lipoprotein receptor‐related protein 1 (LRP1) to achieve BBB crossing and GBM cell targeting and inhibit GBM growth through intracellular translation of PTEN.^[^ ^31^ ^]^

## 4 Challenges and Innovative Strategies in CNS Genetic Therapy

Currently, gene‐drug delivery for CNS diseases involves two main challenges: obstruction of the BBB and low targeting ability. To address these difficulties, researchers have developed and modified carriers to improve the efficiency of gene‐drug delivery in the CNS. In the following, we will elaborate on the current state of research on BBB crossing drug delivery strategies and CNS disease targeting strategies.

### 4.1 Blood‐Brain Barrier Penetration

BBB is a highly selective barrier formed by the endothelial cells, astrocytes, and pericytes. The structural and functional complexity of the BBB plays a crucial role in maintaining the brain's internal environment and ensuring its stability, protecting the brain from external harmful substances.^[^ ^32^ ^]^ However, the protective effect of the BBB also limits the entry of peripheral drugs into the brain parenchyma, thus reducing the efficacy of drugs in CNS disease. Therefore, designing drug carriers that cross the BBB is an important way to improve the efficiency of CNS gene therapy. Currently, the main methods for gene‐drug delivery systems across the BBB are as follows: protein modification, aptamer modification, peptide modification, biomimetic nanovesicle modification and polymeric nanocarriers. In addition, external devices (magnetism and ultrasound) can also facilitate the opening of the BBB thereby increasing the efficiency of drug delivery in the brain. In this section, we will elaborate on these methods for BBB crossing.

#### 4.1.1 Protein Modification

Protein modification mainly exploits the trans‐BBB properties of some viral proteins with CNS invasiveness. For example, researchers have constructed virus‐mimicking nanoparticles (VMN) with BBB‐across functionality by embedding B encephalitis virus proteins into liposome membranes. VMN successfully delivered siGL3 and siPLK1 into GBM cells and achieved knockdown of GL3 and PLK1 genes, resulting in the inhibition of glioma growth.^[^ ^33^ ^]^

#### 4.1.2 Aptamer Modification

Aptamer modification mainly employs protein ligands to bind to the corresponding receptors on the surface of brain microvascular endothelial cells and achieves BBB crossing of gene drugs through receptor‐mediated transcytosis. For example, the transferrin receptor (TfR) is highly expressed on the BBB and promotes brain uptake of blood iron ions by binding ferritin. Based on this characterization, researchers developed a ferritin nanocage (HFn@Fe/siGPX4) that co‐encapsulates iron and siGPX4. Ferritin of HFn@Fe/siGPX4 facilitates BBB crossing and delivery of siGPX4 into GBM cells by recognizing TfR on brain microvascular endothelial cells via transcytosis.^[^ ^34^ ^]^

#### 4.1.3 Peptide Modifications

Peptide modifications can also facilitate BBB crossing of gene drugs. For example, RVG, the surface‐exposed viral capsid protein in viral particles, interacts specifically with the nicotinic acetylcholine receptor (nAchR) thereby crossing the BBB.^[^ ^35^ ^]^ Inspired by this, investigators inserted an RVG‐derived peptide on the surface of macrophage‐derived small extracellular vesicles (sEVs) and synthesized the sEVs^RVG^ gene‐drug delivery system. sEVs^RVG^ inhibits fetal brain damage caused by Zika virus viral infection during pregnancy by loading Zika virus‐specific siRNA (siZIKA).^[^ ^36^ ^]^

#### 4.1.4 Biomimetic Nanovesicle Modification

Biomimetic nanovesicle modification is a method of gene‐drug delivery to the brain by loading drugs into sEVs or vesicles of cell membrane origin. Biomimetic nanovesicles have high biocompatibility, long circulation, and immune escape ability, and thus are ideal carriers for gene drugs.^[^ ^37^ ^]^ The membrane fluidity of biomimetic nanovesicles can also mediate their crossing of the BBB via paracellular and membrane fusion pathways. Therefore, these natural properties of biomimetic nanovesicles make them an ideal tool for gene‐drug delivery for brain diseases. For example, researchers synthesized REXO‐C/ANP/S nanomaterials by co‐loading siSNCA and curcumin into macrophage‐derived sEVs via sonication. REXO‐C/ANP/S exhibited strong BBB permeabilization efficiency and delivered siRNA to the site of cerebral Parkinson's disease and knocked out the relevant genes mediating the production of α‐syn, resulting in the removal of the pathogenic proteins.^[^ ^37^ ^]^

#### 4.1.5 Nanoplatforms Sizes

The size of the nanoplatforms is also an important factor influencing BBB penetration. Nanoparticles (NPs) ranging from 10 to 100 nm are generally regarded as optimal for BBB traversal, as they exibit steady diffusion efficiency and avoid rapid systemic clearance. Smaller NPs (typically 〈20 nm) demonstrate superior diffusion capabilities, either through endothelial gaps or via carrier‐mediated transport. For instance, 20 nm insulin‐coated gold nanoparticles (INS‐GNPs) exhibited superior brain accumulation in murine models compared to their larger counterparts (50–70 nm), owing to efficient insulin receptor‐mediated transcytosis.^[^ ^38^ ^]^ Similary, BSA and MnO_2_ nanocomplexes (5–10 nm) showed remarkable GBM‐specific accumulation within 12 hours of administration.^[^ ^39^ ^]^ For nanomaterials in the 50–200 nm range, BBB penetration predominantly occurs through receptor‐mediated transcytosis following surface modification. A notable example includes glucose‐ and alginate‐functionalized polymeric quantum dots (GT‐PCD) loaded with plasmid DNA (pDNA), which achieved successful brain delivery via glucose transporter protein‐1 (Glut‐1)‐mediated transcytosis, thereby enhancing therapeutic outcomes in PD.^[^ ^40^ ^]^ Conversely, larger NPs (〉200 nm) may exploit alternative pathways such as endothelial macropinocytosis, facilitating internalization of sizable cargo, as evidenced by transport vesicles (〉200 nm) capable of translocating gold nanoparticles into endothelial ceslls.^[^ ^41^ ^]^

#### 4.1.6 External Devices

External devices mainly include magnetic resonance‐guided focused ultrasound (MRgFUS), laser interstitial thermal therapy (LITT), electrical fields and electroporation, convection‐enhanced delivery (CED).^[^ ^42^ ^]^ MRgFUS utilizes ultrasound to transiently disrupt the BBB, usually in combination with microbubbles or nanodroplets, to amplify the cavitation effect. MRgFUS is a non‐invasive precision‐targeting technique guided by magnetic resonance imaging that enables localized and reversible BBB opening (permeability duration ≤ 24 h).^[^ ^43^ ^]^ For example, the investigators constructed a novel perfluoropropane microbubble, termed ApoER‐Pep‐MB, which has a siloxane‐bonded cross‐linked surface to increase the stability of the microbubble against circulating turbulence, and targeted the BBB with a binding peptide for the apolipoprotein E receptor (ApoER‐Pep).In the presence of an external FUS, the BBB was opened within 2 h, dramatically increasing the drug's aggregation in the brain.^[^ ^44^ ^]^ LITT ablates tumors and disrupts the BBB by decreasing the integrity of the tight junctions. LITT prolongs BBB permeability (weeks to months) and is suitable for slow‐release drugs.^[^ ^45^ ^]^ Electric fields and electroporation application of pulsed electric fields to transiently open the BBB through cytoskeletal remodeling of endothelial cells The advantage of electric fields and electroporation is that they are administered non‐invasively through electrodes mounted on the scalp to minimize adverse effects.^[^ ^46^ ^]^ CED is infused directly intracranially through a catheter under positive pressure. The advantage is that the BBB is completely bypassed and high local drug concentrations are achieved.^[^ ^47^ ^]^

### 4.2 Targeting Ability

Off‐target effects of gene drugs are an important factor in systemic side effects. Although gene drugs specifically inhibit or upregulate the expression of target genes, incorrect complementary pairing of some gene bases or low CNS delivery efficiency can trigger unexpected gene silencing or activation.^[^ ^48^ ^]^ Considering these challenges, researchers have made innovative designs and efforts in the targeting of gene drugs aimed at restricting gene drugs to regions of CNS disease or a certain class of cells and organelles (**Table** **2**).

**Table 2:** Targeting Strategies for CNS Disease.

	Targeted Strategies	Gene drugs	CNS diseases	Reference
CNS organ targeting	Neurotransmitter homologous nanomaterials	Cre mRNA, Pten mRNA	GBM	[49]
	Magnetic field	siGPX4	GBM	[50]
	Ultrasound field	IL‐12 mRNA	GBM	[51]
	Lateral ventricular injection	AAV‐MECP2, AAV‐ hfCas13Y	Neurodevelopmental disorder	[52]
	Nasal inhalation	AAV‐CRISPR‐Cas9; HTR2A	Anxiety disorder	[55]
	Deep cervical lymph nodes	‐	‐	‐
Cell targeting	Arc protein shells	GFP mRNA	Chronic neuro‐inflammation	[57]
	Protein ligand	siPKM2	GBM	[59]
		IL‐10 mRNA	Brain injury	[60]
Lysosome	Targeted peptides	‐	‐	‐
	Low pH response	siPLK1	GBM	[63]
Mitochondria	Electric charge	siATP6, siCYB	Brain tumor	[64]
Golgi apparatus	Caveolin‐mediated transcytosis	SiATG7	Brain tumor	[66]

#### 4.2.1 CNS Targeting

Modification of gene delivery carriers using brain chemotactic substances is an important approach to CNS targeting. For example, to improve the efficiency of CNS delivery of mRNA, researchers synthesized levodopa‐derived lipids (LD), D‐serine‐derived lipids (DS), temozolomide‐derived lipids (TM), tryptamine‐derived lipids (TD), cinnamic acid‐derived lipids (CD). These liposomes have neurotransmitter‐like brain chemotaxis that can carry mRNA across the BBB and enable CNS targeting.^[^ ^49^ ^]^

Externally applied fields (e.g., ultrasound fields, magnetic fields, etc.) are another important way to achieve brain targeting of gene drugs. For example, investigators linked Fe_3_O_4_ to siGPX4‐loaded mesenchymal stem cell sEV via CD63 aptamers and synthesized ferroptosis nanocomplex. This nano‐complex aggregated in the brain in the presence of an applied magnetic field, thereby knocking out GPX4 gene and promoting GBM ferroptosis mediated by Fe_3_O_4_.^[^ ^50^ ^]^ In addition to magnetically responsive nanomaterials, ultrasound‐responsive nanomaterials are also an important method for achieving CNS‐targeted gene‐drug delivery. The researchers loaded the mRNA of IL‐12 into calcium carbonate nanoparticles and modified the GL261 cell membrane on the surface. Under the ultrasound field, calcium carbonate nanoparticles can be targeted by generating CO_2_ bubbles in the acidic state of tumor lysosomes, thus achieving the immunocidal effect of GBM via IL‐12 mRNA.^[^ ^51^ ^]^

The targeting of CNS can also be improved by different routes of drug injection. Currently, three effective CNS‐targeted injection routes are being investigated. First, lateral ventricular injection. Lateral ventricular injections can deliver gene drugs directly into the brain, such as brain knockouts or overexpressed lentiviral transfections. This type of injection maximizes the confinement of the gene‐drug to the CNS region, but has the disadvantage of causing puncture damage to the brain tissue and cannot be repeated for multiple injections.^[^ ^52^ ^]^ For example, investigators achieved stable and sustained expression of hfCas13Y and MECP2 gene gRNAs in the brain in a mouse model by lateral ventricular injection of high‐fidelity and highly active adeno‐associated virus (AAV) vectors.^[^ ^53^ ^]^ Second, nasal inhalation. Nasal inhalation allows the drug to be absorbed directly through the olfactory bulb and finally delivered to the brain via the olfactory nerve. The advantage of this delivery method is that it bypasses the BBB obstruction, thus allowing the drug to enter the brain parenchyma directly. However, the small area of the nasal mucosal epithelium limits the amount of drug that can be administered in a single dose.^[^ ^54^ ^]^ For example, Researchers synthesized an AAV‐loaded CRISPR‐Cas9 gene editing system (AAV‐CRISPR‐Cas9) and targeted the HTR2A gene in neurons after nasal inhalation. AAV‐CRISPR‐Cas9 inhibited the expression of the neuronal excitatory neurotransmitter, 5‐hydroxytryptamine receptor, thereby alleviating anxiety symptoms in mice.^[^ ^55^ ^]^ Third, deep cervical lymph node injections. This injection allows the drug to be absorbed by the deep cervical lymph nodes and enter the brain tissue through the meningeal lymphatics. For example, For example, lipid nanoprobes carrying Melitten can be used to target brain tissue via subcutaneous injection in the neck and enable immunomodulation of breast cancer brain metastasis thus inhibiting tumor growth.^[^ ^56^ ^]^ However, gene therapies related to the deep cervical lymph node pathway are currently limited in research, and this is an important direction for future research in CNS diseases.

#### 4.2.2 Cell Targeting

Cells in CNS disease primarily include neurons, tumor cells and glial cells. The cellular targeting of gene‐drug carriers is mainly mediated by specific proteins expressed on the cell surface. In neurons, the Arc proteashell, an infectious RNA retrovirus analog, can mediate interneuronal communication through receptor‐mediated transcytosis. Incorporation of Arc protein shells during cellular sEV synthesis efficiently encapsulates mRNA and enables targeted gene delivery to neurons.^[^ ^57^ ^]^ In GBM cells, some surface proteins are up‐regulated in GBM cells compared to normal brain tissues (such as intercellular cell adhesion molecule‐1 (ICAM‐1),^[^ ^58^ ^]^ TfR,^[^ ^34^ ^]^ and LRP1^[^ ^59^ ^]^). By recognizing these highly expressed protein receptors, gene drugs can be successfully targeted to GBM cells. For example, Investigators designed a nanocapsule co‐encapsulated with temozolomide and siRNA for pyruvate kinase M2 (PKM2) and surface‐modified with ApoE to target LRP1. ApoE‐modified nanocapsules exhibited strong GBM cell recognition and inhibited glycolysis energy production through PKM2 gene knockdown effect thereby promoting chemosensitization of temozolomide.^[^ ^59^ ^]^ In glial cells, the inflammatory or tumor microenvironment causes glial cells to develop an activated state that alters the expression of proteins on their surface. Therefore, this property of glial cells can be utilized for targeted delivery of gene drugs for therapy. For example, in ischemic stroke, M2 microglia highly express mannose receptors. Attachment of mannose to the surface of lipid nanoparticles promotes IL‐10 mRNA targeting to M2 microglia at the site of inflammation and thus repairs brain injury.^[^ ^60^ ^]^

#### 4.2.3 Organelle Targeting

The organelles mainly include lysosomes, mitochondria, endoplasmic reticulum, Golgi apparatus, nucleus, etc. Researchers have developed different targeting strategies for the properties of different organelles.

Lysosomal targeting strategies mainly include the following: (a) Lysosome‐targeted peptides. Tumor cell membrane surfaces express some lysosome‐specific transport proteins, such as the non‐cation‐dependent mannose‐6‐phosphate receptor (CI‐M6PR). Targeted translocation of nanocarriers into lysosomes can be facilitated by the insertion of the corresponding targeting peptide polymannose‐6‐phosphate (M6P).^[^ ^61^ ^]^ (b) Low pH response. The pH range within lysosomes is typically maintained between 3.5 and 5.5.^[^ ^62^ ^]^ This acidic environment is achieved by specific transport proteins in the lysosomal membrane, known as proton pumps. Therefore, some acid‐responsive drug delivery carriers are expected to be tools for lysosomal targeting. For example, tannic acid nano complexes loaded with siPLK1 can degrade and release siRNAs in the acidic environment of tumor cell lysosomes thereby achieving PLK1 gene knockdown in GBM.^[^ ^63^ ^]^

Mitochondria in CNS disease contain some of the mtDNA. Therefore, targeting mitochondrial mtDNA is an effective strategy for CNS gene therapy. Since the inner mitochondrial membrane is negatively charged, positively charged nanocarriers are the main tool for mitochondrial targeting. For example, researchers synthesized nanomaterials carrying the cationic peptide MMPA and encapsulated two siRNAs that regulate mitochondrial DNA‐encoded proteins (siATP6 and siCYB). These mitochondria‐targeted nucleic acid nanoplatforms successfully achieved knockdown of ATP6 and CYB protein expression in brain tumors.^[^ ^64^ ^]^

Targeted gene therapy for endoplasmic reticulum in CNS disease is currently limited in research, so we only describe endoplasmic reticulum‐targeting approaches here. Current approaches to endoplasmic reticulum targeting are mainly by specific ligand modifications such as small molecules and short peptides. For example, Pardaxin‐modified cationic liposomes can accumulate in the endoplasmic reticulum via a non‐lysosomal intracellular pathway. This pathway allows DNA loads to be retained in the endoplasmic reticulum close to the nucleus, thereby facilitating DNA entry into the nucleus and increasing the efficiency of gene therapy.^[^ ^64^ ^]^

Golgi‐targeted therapies are also primarily achieved by targeting peptides or targeting small molecule modifications. Studies have demonstrated that indomethacin (IMC)‐modified small molecule compounds possess Golgi‐specific targeting properties and promote tumor cell death by loading the Golgi structure disrupting agent trans‐retinoic acid (RA).^[^ ^65^ ^]^ In addition, caveolins are major substances in Golgi‐mediated transcytosis, so targeting caveolins can also transport gene drugs to the Golgi apparatus. For example, researchers synthesized a peptide‐polysaccharide nanopolymer for transforming the intracellular delivery pathway of siRNA to exert efficient RNA interference effects. This nanopolymer can initiate caveolin‐mediated transcytosis by recognizing CD44 on the surface of tumor cells to deliver siRNA to the Golgi apparatus.^[^ ^66^ ^]^

## 5 Nanoplatforms for CNS Genetic Therapy

Since siRNA, mRNA, circRNA and CRISPR systems are susceptible to clearance in the circulation, it is often necessary to encapsulate these gene editing drugs in carriers to increase the efficiency of gene knockdown or up‐regulation. Currently, commonly used gene drug carriers mainly include: BNVs, VLPs, liposomes, inorganic nanocarriers. We will elaborate here on the advantages of each of the different vectors in gene therapy for CNS disease (**Table** **3**).

**Table 3:** Nanocarriers for CNS gene therapy.

Nano carriers	Gene drugs	CNS diseases	References
sEV	siPPM1D	Diffuse endogenous pontine glioma	[69]
	siSTAT3	GBM	[70]
	siMYC	GBM	[71]
CMNVS	miR‐9, miR‐21, miR‐215, and miR‐221	GBM	[27]
OMVs	siCD47	GBM	[72]
VLPs	GFP mRNA	Chronic neuro‐inflammation	[57]
Proteins	siEGFR	GBM	[75]
Liposome	m1Ψ mRNA	Autoimmune encephalomyelitis	[77]
	siCD47, siPD‐L1	GBM	[78]
	sic‐myc	GBM	[79]
	CRISPR‐Cas9 system for GFP	GBM	[80]
Polymeric nanocarriers	siCircOGDH	Ischemic stroke	[82]
Mesoporous SiO_2_	Cre‐mRNA	GBM	[83]
MOF	Nrf2‐sgRNA‐guided CRISPR	AD	[84]
Au	siPLK1	GBM	[85]

### 5.1 BNVs‐Mediated CNS Genetic Therapy

BNVs mainly include sEVs, microvesicles, apoptotic bodies (ApoBDs), cell membrane nanovesicles (CMNVs), and cellular nanovesicles (CNVs).^[^ ^52^ ^]^ BNVs are used as ideal carriers for brain gene therapy due to the advantages of high biocompatibility, BBB crossing, and long circulation. Currently, the main BNVs used for CNS gene delivery are sEVs and CMNVs, so in this section, we will describe the gene delivery strategies mediated by these two types of BNVs.

sEV is secreted by cells and has a lipid bilayer structure with a size of 50–150 nm. sEVs are enriched with proteins, lipids, and various nucleic acid molecules and metabolites of the parent cell, and therefore have a wide range of applications in the diagnosis and treatment of diseases.^[^ ^67^ ^]^ The hydrophilic cavity structure of sEV can also be loaded with various sizes of genetic drugs, such as siRNA, mRNA, circRNA and CRISPR systems. Due to the deformability and surface protein expression of sEVs, they can facilitate the crossing of internally carried gene drugs across the BBB via the cellular bypass pathway, receptor‐mediated transcytosis, and the membrane fusion pathway.^[^ ^68^ ^]^ For example, encapsulation of PPM1D siRNA in macrophage‐derived sEVs successfully crossed the BBB and inhibited the growth of diffuse endogenous pontine gliomas by knocking down PPM1D gene expression.^[^ ^69^ ^]^ In addition, sEV also has a lysosomal escape function, thus avoiding the risk of lysosomal degradation of gene drugs. For example, U251 GBM cell‐derived sEVs loaded with siSTAT3 did not co‐localize with lysosomes under confocal microscopy after uptake by U251 cells, suggesting that siSTAT3 successfully escaped from lysosomes and achieved knockdown of STAT3 gene expression.^[^ ^70^ ^]^ Small extracellular vesicles (sEVs) exhibit exceptional biosafety profiles and low immunogenicity, making them ideal nanocarriers for gene therapy applications. For example, Investigators obtained exosomes from mesenchymal stem cells (MSCs) and used them to load Myc siRNA (iExo‐Myc) targeting in situ GBM tumors in mice. Following single‐dose administration, iExo‐Myc demonstrated remarkable pharmacokinetic properties, maintaining detectable fluorescence signals for over 48 h while showing significant tumor‐specific accumulation. No significant organ structural damage was detected in immuno‐immunohistochemical staining of major organs in treated mice, confirming the formulation's outstanding biocompatibility and minimal systemic toxicity.^[^ ^71^ ^]^CMNVs are nanovesicles with only a cell membrane structure, which are usually encapsulated on the surface as modifiers of nanomaterials thereby increasing their biocompatibility and CNS targeting efficiency. CMNVs confers BBB‐crossing properties to nanomaterials, enabling delivery of gene drugs to brain parenchyma. For example, researchers designed a microglial cell membrane‐encapsulated microRNA nanosponge. This nanosponge enhances BBB crossing ability with the encapsulation of BV2 microglia membranes and synergistically adsorbs multiple miRNAs (e.g., miR‐9, miR‐21, miR‐215, and miR‐221) in tumor cells thereby inhibiting the malignant proliferation of GBMs.^[^ ^27^ ^]^

In addition, engineered bacterial outer membrane vesicles (OMVs) demonstrate dual therapeutic capabilities by crossing the blood‐brain barrier (BBB) while simultaneously stimulating anti‐tumor immunity. In a representative study, researchers reduced endotoxin levels in OMVs by genetically engineering the knockout of the msbB gene in Escherichia coli (E. coliBL21). These optimized OMVs were then used to co‐deliver doxorubicin (DOX) and CD47 small interfering RNA (siCd47), which effectively reduced the phagocytosis escape ability of GBM cells, thereby enhancing the phagocytosis of GBM and overcoming the acquired immune resistance of GBM cells.^[^ ^72^ ^]^

### 5.2 VLPs‐Mediated CNS Genetic Therapy

VLPs are self‐assembled particulate structures made of viral capsid proteins that have the appearance and function of viral particles but do not contain viral genetic material. This means that VLPs are able to efficiently deliver biomolecules such as mRNA, proteins, while avoiding the safety risks that can be associated with traditional viral vectors.^[^ ^73^ ^]^ Some specific viral protein particles have the ability to induce mRNA loading and CNS targeting. The Arc proteashell, an infectious RNA retrovirus analog that automatically encapsulates mRNA in exosomes during cellular exocytosis and forms the proteocapsid structure of the exosome. This protein‐shell structure can facilitate the transfer of exosomes encapsulated with mRNA between neurons so that the corresponding proteins are expressed translationally in the neuron.^[^ ^57^ ^]^

### 5.3 Protein Nano Drug Delivery Platform

Some natural proteins exhibit hollow spherical architectures and can be subjected to a pH‐mediated reversible disassembly/assembly process, endowing them with ideal characteristics for drug delivery.^[^ ^74^ ^]^ Notably, specific endogenous proteins can facilitate blood‐brain barrier (BBB) penetration through interactions with cell surface receptors. For example, a series of ferritin variants with positively charged cavities and truncated C‐termini, termed tHFn(+), have been designed to take advantage of the inherent tumor‐targeting properties of human H‐ferritin and its ability to traverse the BBB. These nanocarriers are responsive to weak acids and disintegrate in endosomal compartments, exposing the positive charge inside to facilitate the escape of loaded small interfering RNA (siRNA) from the lysosome. As a pervasive siRNA nanocarrier, tHFn(+) significantly enhanced the uptake of different siRNAs and suppressed the expression of genes associated with GBM progression. In addition, tHFn(+) successfully crossed the BBB and targeted gliomas by binding to its receptors, such as transferrin receptor 1.^[^ ^75^ ^]^

### 5.4 Liposome‐Mediated CNS Genetic Therapy

Liposome typically has four components: cationic or ionized lipids, cholesterol, auxiliary lipids, and polyethylene glycolated lipids. Liposomes have a hydrophilic cavity structure so they can be loaded with gene drugs. After liposomes are endocytosed by the cell, the ionizable cationic lipids are protonated to become positively charged in the lower pH endosomes, making their membrane structure unstable and thus rupturing to release the drug.^[^ ^76^ ^]^ Liposomes are used in CNS gene therapy in the following ways: (a) Liposomal mRNA vaccine. Researchers synthesized a non‐inflammatory mRNA vaccine for autoimmune encephalomyelitis by substituting 1‐methylpseudouridine (m1Ψ) for m1Ψ in mRNA uracil (U) and encapsulating it in liposomes. This mRNA vaccine can safely and effectively deliver self‐antigens to antigen‐presenting cells (APCs), allowing the immune system to restore self‐recognition and thus achieve self‐antigen‐specific immune tolerance;^[^ ^77^ ^]^ (b) Gene silencing. This effect is achieved by encapsulating siRNAs in liposomes that can knock down and regulate the expression of target genes. For example, researchers Loaded siCD47 and siPD‐L1 in disulfide bond‐modified ionizable cationic lipids. The BBB‐crossing efficiency of cationic liposomes was enhanced by the mediation of electronegativity on the surface of cerebral microvascular endothelial cells, which led to enhanced drug penetration at the GBM site and facilitated the knockdown of CD47 and PD‐L1 gene expression;^[^ ^78^ ^]^ Recent research has demonstrated the effectiveness of penetratin‐modified liposomes as nasal delivery vehicles for c‐Myc‐targeting siRNA to the brain. Investigators constructed a nucleoshell structural gene delivery system for transnasal entry into the brain by mixing c‐Myc‐antagonizing siRNA with the cationic peptide 8 polyarginine to form an electrostatic complex, which was then encapsulated in cationic liposomes and then surface‐modified with a preferred Penetratin‐derived peptide by a postmodification method. In vivo pharmacodynamic assessments revealed that this nasal delivery system successfully suppressed c‐Myc expression in intracranial tumors, enhanced tumor cell apoptosis, and significantly extended the median survival of glioblastoma‐bearing animals.^[^ ^79^ ^]^ (c) Gene editing for CNS disease by introducing the CRISPR‐Cas9 system in liposomes. For example, a CRISPR‐Cas9 system loaded with GFP gene‐guiding RNA in liposomes successfully achieved knockdown of the GFP gene in glioblastoma stem cells.^[^ ^80^ ^]^

### 5.5 Polymeric Nanocarriers

Polymeric nanocarriers exhibit distinct biological advantages for drug delivery. Their superior biocompatibility and biodegradability compared to inorganic materials address critical concerns regarding systemic toxicity and long‐term accumulation. Polymers such as poly(lactic‐*co*‐glycolic acid) (PLGA), polycaprolactone (PCL), and chitosan allow precise control over size (optimized range: 50–150 nm), morphology (rod‐like nanoparticles show higher BBB permeability than spherical ones due to increased adhesion), and surface charge (negative or neutral charges minimize protein opsonization and prolong circulation time).^[^ ^81^ ^]^ Surface functionalization with ligands (e.g., transferrin, lactoferrin, angiopep‐2) enables receptor‐mediated transcytosis, leveraging BBB‐expressed receptors for targeted delivery. For instance, the investigators loaded CircOGDH siRNA into PLGA and synthesized PLGA@CircOGDHsiRNA nanoparticles. PLGA@CircOGDHsiRNA can cross the BBB and deliver siRNA to the ischemic semi‐dark band region and attenuate neuronal apoptosis in ischemic stroke mice.^[^ ^82^ ^]^

### 5.6 Inorganic Nanomaterial‐Mediated CNS Genetic Therapy

Mesoporous SiO_2_‐based inorganic nanomaterials are the most common gene therapy carriers due to their large internal drug‐carrying space and stable chemical structure. By attaching disulfide bond‐linked targeting peptides to mesoporous SiO_2_ surfaces, researchers successfully constructed GSH‐responsive nanogene delivery capsules. This nanocapsule can be loaded with DNA, mRNA and CRISPR gene editor, and successfully achieved knockdown and editing of GBM target genes.^[^ ^83^ ^]^

Metal‐organic framework (MOF) are formed by self‐assembly of inorganic metal centers (metal ions/clusters) and organic ligands via coordination bonds, with periodic network structure, exhibiting high specific surface area, ultra‐high porosity, and structural tunability. The cavity structure of MOF is also functional for gene drug loading. For example, The researchers loaded the Nrf2‐sgRNA‐guided CRISPR activation system into the MOF structure and modified the peptide K8 on its surface to cross the BBB. This MOF‐based gene therapy system successfully achieved activation of Nrf2 and expression of downstream redox genes, which effectively alleviated cognitive impairment in Alzheimer's disease mice.^[^ ^84^ ^]^

Aurum (Au) nanomaterials, particularly Au nanosheets (AuNSs) exhibit exceptional properties for combined photothermal‐gene therapy in brain diseases, including an extensive modifiable surface area, outstanding photothermal conversion efficiency (>80%), and remarkable thermal stability. A prime example is the development of RGD peptide‐ and siRNA‐functionalized Au nanosheets (AuNSs‐RGD‐C≡C‐siRNA, ARCR) for multimodal glioblastoma treatment. It was found that the alkyne group could be assembled to the surface of Au within 1 min, allowing siRNA to form strong covalent binding with AuNSs, which could avoid the interference of biothiols. In addition to the convenience of modification, the photothermal effect of Au nanosheets enhanced the therapeutic effect of siPLK1 and promoted apoptosis in GBM cells.^[^ ^85^ ^]^

## 6 Applications of Nanogenetic Therapy Platforms in CNS Diseases

**Figure 2:**
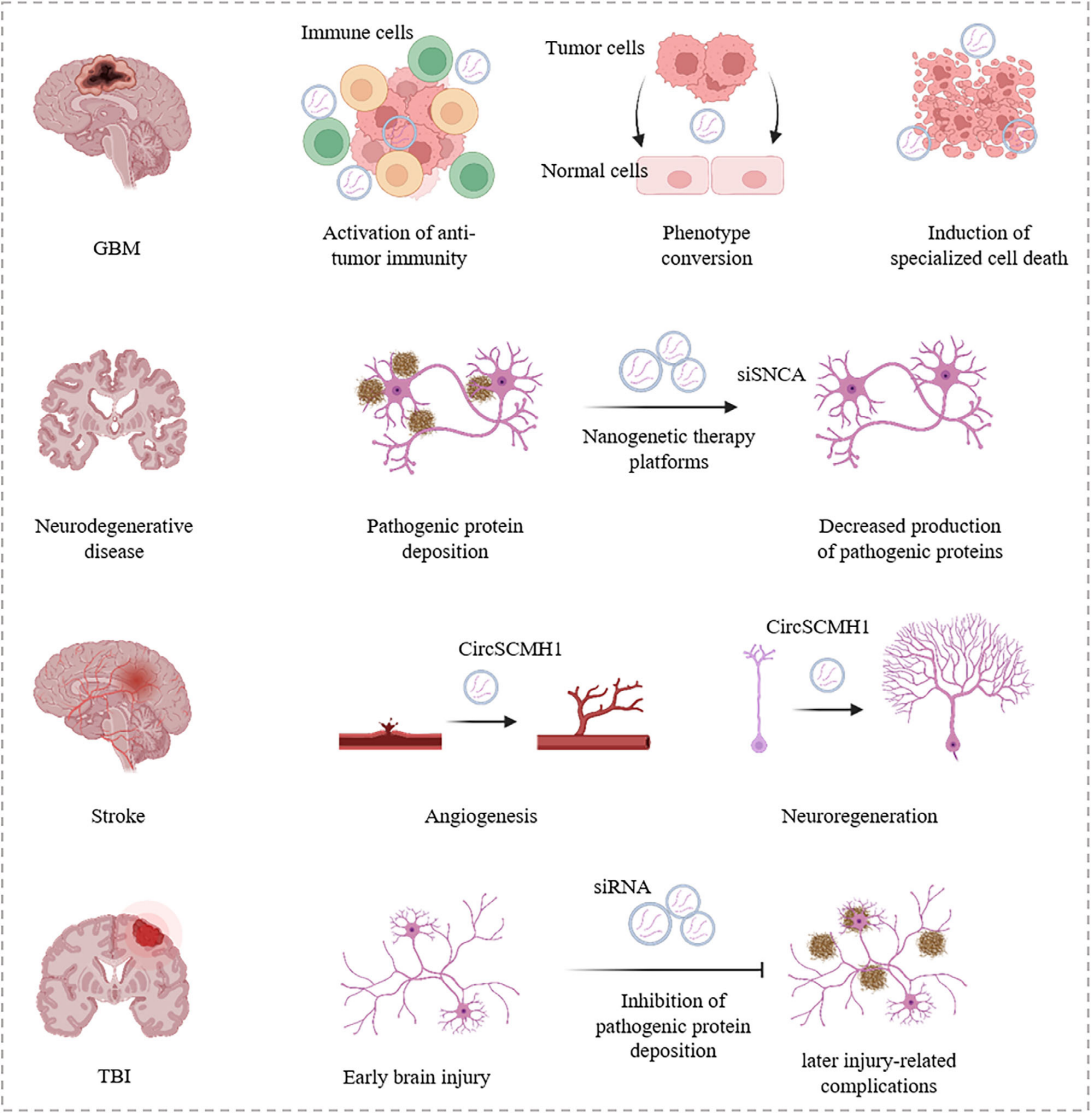
Applications of nanogenetic therapy platforms in GBM, neurodegenerative diseases, stroke and TBI. Nanogenetic therapy platforms activate anti‐tumor immunity, promote phenotypic transformation of tumor cells, and induce specific cell death modalities in GBM; inhibit the production and accumulation of therapeutic proteins in neurodegenerative diseases; promote angiogenesis and neural regeneration in stroke; and reduce and prevent later injury‐related complications in TBI.

CNS diseases primarily include GBM, neurodegenerative diseases, ischemic stroke and traumatic brain injury. Mechanistically, GBM arises from genetic mutations in normal cells,^[^ ^86^ ^]^ and genetic mutations in neurodegenerative diseases can also lead to excessive accumulation of pathological proteins.^[^ ^87^ ^]^ Therefore, these two diseases are the main areas of application for CNS gene therapy. Ischemic stroke and traumatic brain injury are acute in onset, and gene therapy is difficult to perform in the early stages of the disease due to the long time required for gene overexpression or knockdown. Therefore, the main applications of gene therapy for ischemic stroke and traumatic brain injury are in the later stages to promote neural repair and vascular regeneration. Here, we will present the application of nanogenetic therapy in CNS diseases (**Figure** **2**).

### 6.1 GBM

GBM is an extremely malignant brain tumor with a median survival of only 15 months. A large number of mutated and overexpressed genes are present in GBM, so gene therapy can be used to inhibit GBM growth. Gene therapy with GBM can produce a variety of tumouricidal effects, for example, activation of anti‐tumor immunity, and inhibition of angiogenesis. Next, we will focus on these tumoricidal effects of gene therapy in GBM. (a) Activation of anti‐tumor immunity. Researchers used GBM cell‐derived sEV as a GBM‐targeted delivery carrier and loaded the CD47 gene editing system and IL‐9 overexpression plasmid in it. This gene therapy nanomaterial achieves knockdown of CD47 and overexpression of IL‐9 in GBM cells, thus promoting both phagocytoses of tumors by microglia and specific killing of GBM by T cells.^[^ ^88^ ^]^ (b) Phenotype conversion. Differentiation of GBM cells to normal neuronal cells by genetic editing, therefore inhibiting the malignant growth of GBMs. For example, GBM cells display neuronal morphology and express immature neuronal markers after overexpression of the neuro transcription factors Neurog2 or NeuroD1 by viral transfection, thereby slowing the growth of GBM cells.^[^ ^89^ ^]^ (c) Induction of specialized cell death. Investigators achieved iron ion release and knockdown of the ferroptotic protective gene GPX4 by combining siGPX4 and Fe_3_O_4_ in the same nanocomplex, thereby promoting ferroptosis in GBM cells.^[^ ^34^ ^]^ (d) Anti‐angiogenesis. In a previous study, siVEGFR2 was loaded into the polymer PEG‐b‐P (Gu/Hb) and self‐assembled into GBM‐targeted nanomaterials. This gene therapy nanomaterial mediated the knockdown of VEGFR2 in GBM and reduced GBM angiogenesis, resulting in tumor growth inhibition.^[^ ^90^ ^]^

### 6.2 Neurodegenerative Disease

Neurodegenerative diseases mainly include AD and PD. The main pathological change in AD and PD is the accumulation of pathogenic proteins in nerve cells. In neurodegenerative diseases, gene mutations in nerve cells are important factors leading to the production of pathological proteins, for example, SNCA gene mutation. SNCA is the main gene encoding α‐synuclein, and mutations in it can lead to abnormal accumulation and misfolding of α‐synuclein, thereby accelerating the pathological process of PD.^[^ ^91^ ^]^ Thus, targeting mutant genes significantly inhibits the production of pathogenic proteins and thus improves the prognosis of neurodegenerative diseases. For example, nano scavengers synthesized by loading siSNCA into macrophage‐derived sEVs reduced α‐synuclein production and alleviated symptoms of PD in mice by targeting knockout of the SNCA mutant gene.^[^ ^92^ ^]^ In addition to the removal of pathogenic proteins, gene therapy to promote nerve regeneration is also promising in the treatment of neurodegenerative diseases. Researchers transfected AAV loaded with the NeuroD1 gene into the brain via orbital injection. NeuroD1 mediates astrocyte to neuron differentiation and replacement of inactivated neurons, which ameliorates cognitive deficits in AD.^[^ ^93^ ^]^

### 6.3 Stroke

Ischemic Stroke, as a common cerebrovascular disease, is the leading cause of disability and death in adults in China. Gene therapy for ischemic stroke involves angiogenesis and neuroregeneration.

Angiogenesis promotes energy and oxygen supply to brain tissue and is therefore a key factor in the recovery of neurological function in ischemic stroke. 293T cell‐derived sEV carrying circSCMH1 can target ischemic stroke sites via RVG peptides. CircSCMH1 promotes ubiquitination modification of fat mass and obesity‐associated protein (FTO) in cerebrovascular endothelial cells and increases FTO translocation to the nucleus, leading to a decrease in the m6A modification of lipid phosphate phosphatase 3 (Plpp3), a gene related to vascular development. This effect inhibited the degradation of Plpp3 mRNA by YTHDF2, leading to increased levels of Plpp3 mRNA with its encoded protein LPP3 in endothelial cells and promoting vascular repair.^[^ ^94^ ^]^

Since there is a large amount of neuronal necrosis in ischemic strokes that results in impaired neurological function, the promotion of neural regeneration is also an important measure for the repair of brain damage. Investigators obtained sEVs carrying circSCMH1 and RVG modifications (RVG‐circSCMH1‐EV) by transfection of 293T cells. RVG‐circSCMH1‐EV treatment increased the number of spine columns, neuronal synapse branching, dendritic length, and neuronal crossings in peri‐infarcted cortex of PT mice at 28 days postoperatively.^[^ ^29^ ^]^

### 6.4 Traumatic Brain Injury

Gene therapy for traumatic brain injury is currently focused on suppressing late complications. The tau proteins are microtubule‐associated proteins closely associated with brain injury‐related chronic traumatic encephalopathy and traumatic Alzheimer's disease. Thus modulation of tau accumulation after traumatic brain injury is a key approach to suppressing late complications. Researchers loaded siRNAs of tau proteins in PS 80 nanoparticles and modified the surface with PEG and the cross‐BBB ligand Tf. This gene nano‐delivery platform inhibited the accumulation of tau protein in the brain after traumatic brain injury, thereby reducing and preventing later injury‐related complications.^[^ ^95^ ^]^

## 7 Clinical Translation Prospects

The clinical translation of nano‐enabled gene therapies has achieved significant milestones, propelled by synergistic advancements in nanodelivery platforms (e.g., lipid nanoparticles (LNPs), engineered viral vectors and precision genome‐editing technologies like CRISPR/Cas9). Notably, Wang ’s group developed low‐immunogenic LNPs to effectively deliver PD‐L1 mRNA, successfully inducing tolerogenic antigen‐presenting cells (tol‐APCs) in vivo. This strategy suppressed pathogenic T cells and expanded regulatory T cells, demonstrating efficacy comparable to TNF‐α inhibitors but with reduced systemic toxicity and cost (1% of traditional cell therapies).^[^ ^100^ ^]^ Despite these breakthroughs, long‐term biodistribution and genotoxicity data for nanocarriers remain sparse. Regulatory agencies require comprehensive preclinical profiling to mitigate risks, slowing clinical translation. In addition, the ethical debate around heritable gene modifications, as highlighted in discussions on germline therapy, remains a critical barrier. Unintended consequences, such as “gene elitism” or unintended off‐target effects, raise fears of irreversible societal inequities. The convergence of nano‐gene therapy with complementary treatment modalities (immunotherapy and small‐molecule drugs) represents a paradigm shift in overcoming therapeutic resistance and achieving enhanced antitumor responses.^[^ ^101^ ^]^ This integrated approach capitalizes on the unique capabilities of nanocarrier systems ‐ including tissue‐specific targeting, spatiotemporal control of drug release, and minimized off‐target effects ‐ to create synergistic therapeutic platforms that simultaneously modulate gene expression, immune responses, and metabolic pathways. A compelling demonstration of this strategy involves the co‐delivery of DOX and CD47‐targeting siRNA (siCD47) using engineered bacterial OMVs. In this system, DOX enhances the immunogenicity of GBM by inducing immunogenic cell death (ICD), while siCD47 inhibits CD47 expression to promote phagocytosis of tumor cells by macrophages, which together enhance the immunocidal effect of GBM.^[^ ^72^ ^]^

## 8 Discussion and Conclusion

Mutations or abnormal epigenetic modifications of genes are important factors in the development and progression of CNS disease. Some genetic drugs (e.g., siRNAs, miRNAs, mRNA, circRNA, CRISPR) are effective in inhibiting these pathological changes, with promising applications in CNS disorders. However, due to the obstruction of BBB, gene drugs are difficult to aggregate in the brain thereby reducing the efficacy of gene therapy for CNS diseases. Modifiability of nanoplatforms (mainly including protein modification, aptamer modification, peptide modification, biomimetic nanovesicle modification), size effect of nanomaterials and external devices can positively affect the BBB crossing efficiency of drugs. These approaches allow gene drugs to enter the brain parenchyma by opening the BBB either via active mechanisms (receptor‐mediated transcytosis) or passive routes (passive diffusion through intercellular pathways). Nevertheless, several limitations must be addressed: (a) individual differences in BBB integrity and transporter receptor expression may result in treatment response heterogeneity;^[^ ^96^ ^]^ (b) Potential neurotoxicity concerns arise from prolonged nanoparticle accumulation in cerebral tissues;^[^ ^97^ ^]^ (c) External device‐induced BBB disruption, whether transient or sustained, may impact neurological function;^[^ ^98^ ^]^ and (d) Disease‐specific BBB permeability (e.g., disrupted BBB in stroke/TBI versus relatively intact barriers in neurodegenerative disorders/GBM) necessitate tailored nanomaterial design strategies to account for pathological heterogeneity.^[^ ^43^, ^96^, ^99^ ^]^ These considerations must be comprehensively addressed in the next generation of BBB‐traversing nanotherapeutic development.

Nanoplatform‐based genetic therapy has emerged as a transformative paradigm for CNS therapeutics, overcoming the longstanding challenge of blood‐brain barrier (BBB) penetration while achieving unprecedented spatial and temporal control of therapeutic delivery. Future efforts should focus on optimizing combinatorial regimens, advancing clinical trials, and addressing ethical and regulatory hurdles to achieve the full potential of these synergistic therapies. Addressing these challenges will be critical to bridge the translational gap for these innovative nanotherapeutics, paving the way for paradigm‐shifting treatments for refractory CNS disorders.

## Conflict of Interest

The authors declare no conflict of interest.
